# Shear bond strength of debonded ceramic restorations re-cemented by means of a cleaning and retreatment protocol

**DOI:** 10.4317/jced.55706

**Published:** 2019-06-01

**Authors:** Luisa Pineda-Vásquez, Antonio Fons-Font, Jose-Luis Bustos-Salvador, Jorge Alonso-Pérez-Barquero, Juan-Luis Román-Rodríguez

**Affiliations:** 1 DDS, Lecturer in Prosthodontics. Department of Dental Medicine, Prosthodontic and Occlusion Teaching Unit, University of Valencia General Studies (UVGS), Spain; 2 DDS, PhD, MD, Senior Lecturer, Department of Dental Medicine, Prosthodontic and Occlusion Teaching Unit, UVGS, Spain; 3 DDS, MSc, PhD, Associate Lecturer, Department of Dental Medicine, Prosthodontic and Occlusion Teaching Unit, UVGS, Spain

## Abstract

**Background:**

As there is no standard method for re-cementing debonded partial ceramic restorations, the aim of this study was to evaluate the use of a non-invasive thermal protocol for cleaning and retreatment, and to study its influence on shear bond strength.

**Material and Methods:**

Twenty ceramic samples (IPS e.max CAD®) were bonded to composite cement cylinders and underwent a shear bond strength test (G1, n=20). A second group was created (G2, n=20), representing debonded restorations. To simulate debonding, the samples were artificially contaminated with composite cement. After debonding, these underwent a thermal protocol to remove remaining adhesive. After rebonding to the composite cement cylinders, samples underwent the shear bond strength test.

**Results:**

Median bond strengths for G1 and G2 were 7.28±3.23; 7.06±3.41 MPa, respectively, without significant difference between the groups (*p*=0.983).

**Conclusions:**

Debonded lithium disilicate glass-ceramic restorations should undergo a laboratory cleaning and retreatment protocol before being returned to the clinic for rebonding.

** Key words:**Ceramic, adhesive debonding, shear bond strength, porcelain laminated veneers.

## Introduction

In recent years, the field of prosthodontic dentistry has undergone a change in its approach to treatment planning. This has been caused by the increasing socially driven demand by patients for treatments that provide good esthetic outcomes, together with the ongoing objective of preserving dental structures as far as possible. This means performing minimally invasive treatments, which often take the form of partial coverage restorations (1). These must be bonded to teeth following the classic principles of adhesion (2). The long-term success of bonded partial restorations depends on, among other factors, an effective adhesive system (3-5). It has been shown that one of the most common causes of failure for this type of restoration is debonding (6-8). Even when adhesive systems are applied with great care, some 5.5-9% of partial ceramic restorations are seen to debond (8,9).

When a restoration debonds from the tooth due to an adhesive failure and survives intact, the resin adhesive remains adhered to the internal surface of the restoration and must be removed before the conventional bonding procedure can be repeated (5,10,11). In this situation, it must be decided how to remove the adhesive remnant from the restoration and how to create the best conditions for rebonding.

**Figure 1:**
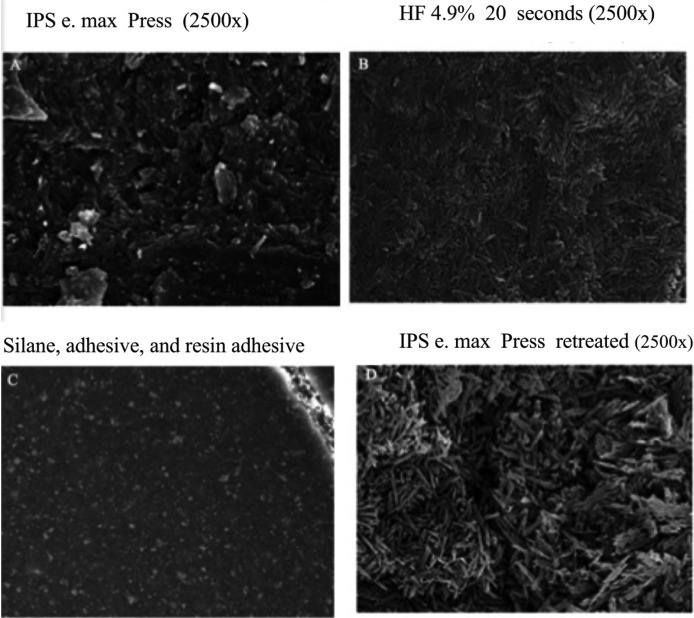
Electronic microscope images of lithium disilicate ceramic surface (A). Change produced by acid etching (B). Ceramic surface after silanizing and applyng resin adhesive (C). Surface almost identical to image B after decontaming surfaces using proposed protocol (D).

In 2015, a protocol was proposed for carrying out decontamination of the ceramic restoration of all resin adhesive remnants, monitoring the technique under electronic microscopy (11) (Fig. 1). Having corroborated the technique under the microscope, it is now necessary to validate the protocol by means of testing shear bond strength.

So, this study aimed to analyze shear bond strength of the ceramic-resin adhesive bond of debonded dental restorations after retreatment and re-cementation.

The null hypothesis was that there would no significant differences in shear bond strength between the two groups assayed, in other words, that the retreatment of debonded restorations would not have a negative effect on bond strength.

## Material and Methods

Forty cube-shaped lithium disilicate glass ceramic (IPS e.max CAD®, Ivoclar Vivadent) samples were fabricated measuring 6x6x6 mm. These were treated according to a conventional bonding protocol ( Table 1), which consisted of etching with 4.9% hydrofluoric acid (HF) (IPS Ceramic Etching Gel®) for 20 seconds, followed by washing in abundant water, and drying. Afterwards, Monobond Plus® universal primer was applied, left to act for 1 minute, and then dried. Lastly, a layer of Excite® was applied without polymerization. The sample of forty specimens was divided into two groups: Group 1 (n=20) and Group 2 (n=20).

**Table 1:**
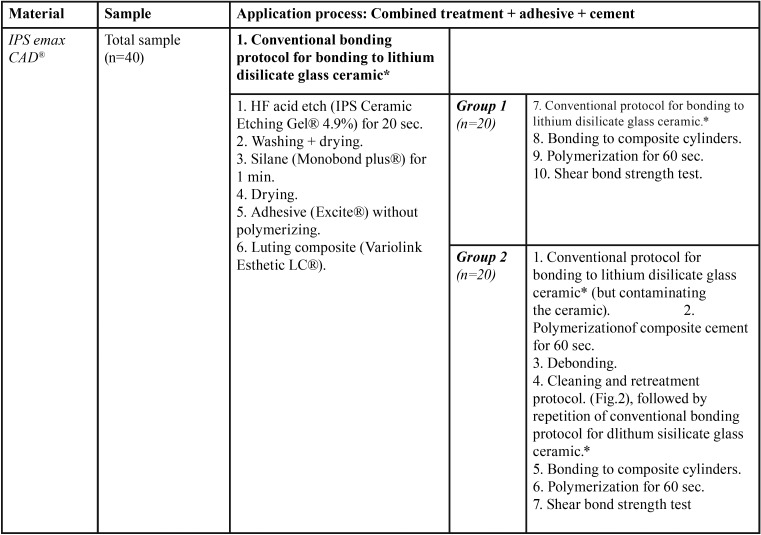
Order of procedures followed in Group 1 (control) and Group 2 (test).

After following the conventional bonding procedure for lithium disilicate glass ceramic, Group 1 (control), 20 previously polymerized composite cement cylinders (Variolink Esthetic LC® Ivoclar-Vivadent) were bonded to the treated surface, using the same photopolymerizable resin adhesive as bonding agent. At this point, excess adhesive was removed and polymerization was applied for 60 seconds. Lastly, the group’s shear bond strength was tested ( Table 1).

**Figure 2:**
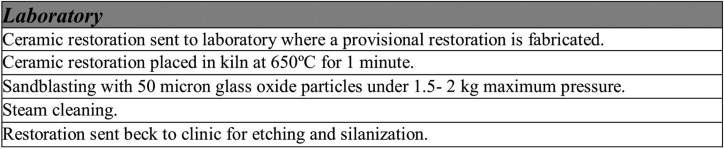
Cleaning and retreatment protocol for debonded restorations, published by Román-Rodríguez in 2015.

To simulate debonded restorations, Group 2 (test) samples underwent the same lithium disilicate glass-ceramic bonding procedure as Group 1, polymerizing the Variolink Esthetic LC® composite cement for 60 seconds in the same way. After debonding the samples, the cleaning and retreatment protocol was applied. This consisted of placing the samples in a kiln at a temperature of 650ºC for one minute, in order to pyrolize the composite prior to retreatment ( Table 1) (Fig. 2). Then, having cleaned the ceramic surface, the conventional bonding procedure was repeated. When complete, the 20 samples underwent shear bond strength testing.

**Figure 3:**
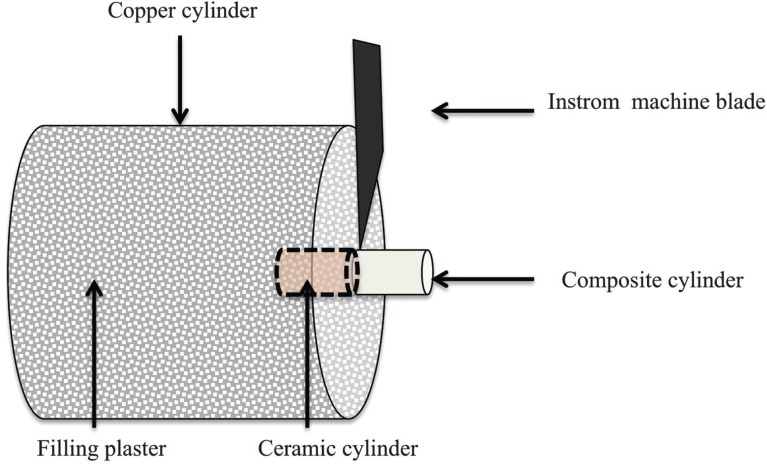
Illustration of sample set in copper cylinder for shear bond testing.

The shear bond test was performed with a Shimadzu AG-X plus universal test machine (Shimadzu Corporation, Kyoto, Japan) with 1000 N load cell. All samples were set in copper cylinders using Type IV plaster. The cylinders were positioned firmly and horizontally in the test machine (Fig. 3). The machine applied force to the composite cylinder until the moment when fracture of the cement-restoration ensemble was produced.

To compare distribution of force values between groups, descriptive statistics were calculated: mean, standard deviation, minimum, maximum, and median. Due to the small sample size, non-parametric tests were used (Mann-Whitney). Statistical significance was set at *p*<0.05.

## Results

**Figure 4:**
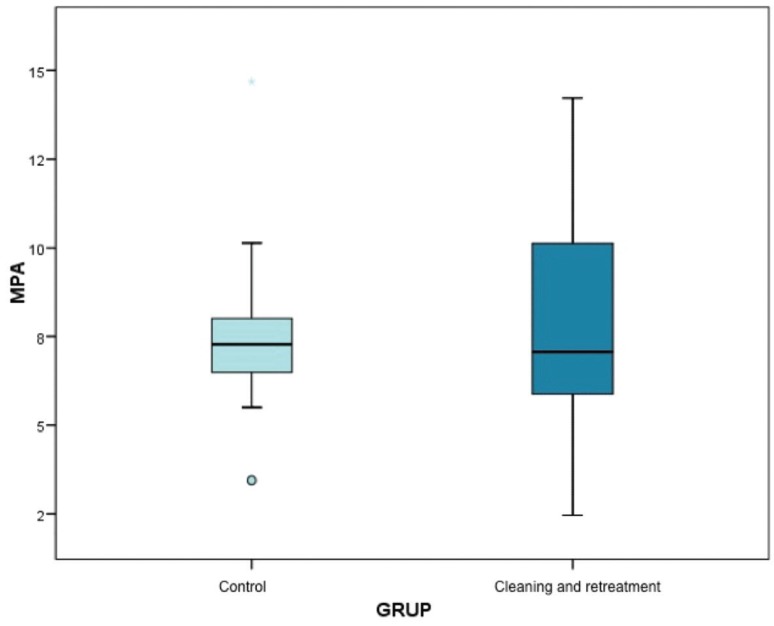
Blox-Pot representating data distribution and median values obtained.

The descriptive statistics calculated were very similar for both groups. The box-plot in Figure 4 represents these data showing the distribution of values obtained. The median force in both groups is situated at almost the same level. Nevertheless, the greater variability in Group 2 is due to the disparate results obtained by two individual samples. The Mann Whitney test confirmed that there was sufficient statistical evidence to conclude that shear bond strength was similar between groups, without statistically significant difference (*p*=0.983), ( Table 2).

**Table 2:**
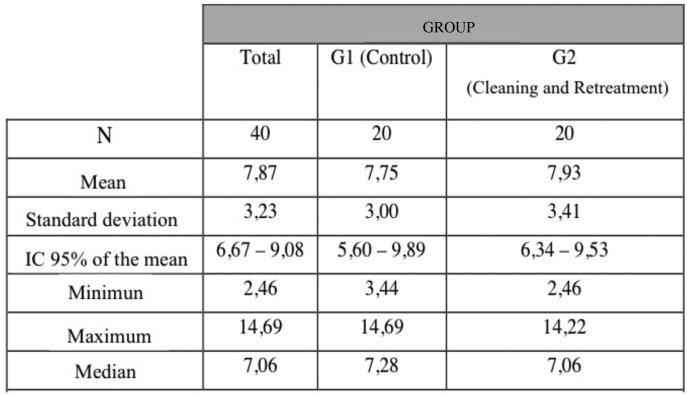
Shows the descriptive statistics for bond strength data (MPa) obtained in both groups. Median bond strength for Group 1 (control) and Group 2 were 7.28±3.23 and 7.06±3.41 MPa, respectively.

## Discussion

The surface pattern created when glass ceramics are etched with hydrofluoric acid varies in relation to the ceramic’s composition and the etching technique used. After etching, both conventional feldspathic and high strength ceramics gain highly retentive surfaces suited to adhesion (12-14). This provides an ideal substrate, which, together with the chemical treatment of the restoration, will obtain predictable bond strengths. For this reason, when a partial restoration debonds, the technique used for cleaning the adhesive remnant should aim to restore the surface’s retentive capacity.

The classic clinical procedure is to remove the resin adhesive from the ceramic restoration’s interior by grinding, followed by sandblasting, re-etching with hydrofluoric acid, resilanization, and rebonding (3). The disadvantage of this procedure is the difficulty of differentiating between adhesive and ceramic, as they have a similar tone and color. If grinding and sandblasting fail to distinguish between the materials, a part of the restoration’s surface may be eliminated resulting in a badly fitting restoration. But, if not all the adhesive is removed, the hydrofluoric acid will fail to etch adequately and fail to create the micro-roughness required.

The literature recommends different methods for removing composite cement. For example, the use of laser technology has been proposed (Er,Cr:YSGG; Er:YAG) for cleaning debonded orthodontic brackets, a technique that has been shown to be effective, and produces bond strength values that are similar to the original bond (15,16). Other methods include sandblasting or silanization followed by an application of silane that produces bond strengths similar to normally bonded brackets (5,17).

A study published by Silva uses an additional thermal treatment, using hot air at 100ºC for 60 seconds applied to previously silanized debonded brackets. The results obtained showed that the shear bond strength achieved was even higher than the original bond (18).

Another technique proposes the use of a smelting or porcelain kiln (5,11,19,20). This consists of placing the debonded restoration in a kiln at a temperature below the ceramic’s melting temperature (650° C) for 1 minute. This burns the remaining resin adhesive before carrying out the conventional mechanical (HF acid etch) and chemical (silanization, adhesive, and bonding) processes. Scanning electron microscope (SEM) images (Fig. 1) show that after applying thermal treatment to the debonded restoration, the surface is left clean and retentive (11).

Analyzing the results of shear bond strength testing, median values in Group 1 and Group 2 were almost identical (7.28 ±3.23 MPa; 7.06 ±3.41 MPa). So, there was sufficient statistical evidence to affirm that the bond strengths obtained in the two groups were very similar (*p*=0.983). Nevertheless, it is clear that Group 1 (control group) showed a more homogenous distribution than the Group 2 (test group). These differences can be attributed to the fact that after eliminating the remains of resin composite, small invisible traces remained in some specific marginal areas, which could have reduced surface adhesion. Similar results to the present study were obtained by St Germain, who analyzed the effect on shear bond strength produced at the enamel-restoration interface of leucite-reinforced ceramic heated to 458º C for 10 minutes; mean bond strength values between test group and control group were not significantly different (11).

## Conclusions

Debonded lithium disilicate glass-ceramic restorations should undergo a laboratory cleaning and retreatment protocol before being returned to the clinic for rebonding.
